# A cross-sectional study on the effects of intravesical BCG on urinary microbiota in bladder cancer patients

**DOI:** 10.1007/s11255-025-04607-x

**Published:** 2025-06-30

**Authors:** Joseph Kai Man LI, Lin WANG, Ryan Tsz Hei Tse, Chi Ho Leung, Kang Liu, Hongda Zhao, Carol Ka Lo Cheng, Christine Yim Ping WonG, Stephen Kwok Wing Tsui, Gianluca Giannarini, Alex Qinyang Liu, Peter Ka Fung Chiu, Chi Fai Ng, Jeremy Yuen Chun Teoh

**Affiliations:** 1 Department of Surgery, S.H. Ho Urology Centre, Prince of Wales Hospital, The Chinese University of Hong Kong, Hong Kong, China; 2 Institute of Trauma and Metabolism, Zhengzhou Central Hospital Affiliated to Zhengzhou University, Zhengzhou, China; 3 School of Biomedical Science, The Chinese University of Hong Kong, Hong Kong, China; 4 Urology Unit, Santa Maria Della Misericordia Academic Medical Centre, Udine, Italy

**Keywords:** Urinary bladder cancer, Urinary microbiome, Mycobacterium bovis Bacillus Calmette–Guérin (BCG), Bacteria, Biosynthesis pathways

## Abstract

**Background:**

Urinary bladder cancer is among the most common malignancy worldwide. Despite surgical interventions and regular surveillance, recurrence and progression to advanced disease are observed in patients. Intravesical administration of Bacillus Calmette–Guérin (BCG) could reduce bladder cancer recurrence and progression in patients with intermediate- and high-risk non-muscle-invasive bladder cancer (NMIBC). Nonetheless, not all patients respond well to BCG treatment. We aim to evaluate whether bacteria profiles were altered after BCG administration.

**Methods:**

In this cross-sectional study, we investigate the differences in urinary microbiome between non-cancerous controls, bladder cancer patients undergoing surveillance cystoscopy, and patients with BCG administration (post-BCG). The V4 regions of the 16S rRNA gene were sequenced, and alpha-diversity and beta-diversity were analyzed. Taxonomic differences between groups and metabolic function analysis of bacteria groups were investigated.

**Results:**

Comparing to the other two groups, the proportion of Pseudomonas, Lactococcus, and Bacillus were increased in the post-BCG group. Superpathways of L-phenylalanine biosynthesis, L-tyrosine biosynthesis, ubiquinol-7, 8, 9, 10 biosynthesis, lipopolysaccharide biosynthesis, glucose degradation oxidative, and 4-hydroxyphenylacetate degradation were significantly enhanced in the post-BCG group.

**Conclusion:**

Results demonstrated that urinary bacteria profiles were distinguished between controls and post-BCG patients. Certain bacteria genus was shown to enhance in post-BCG patients, revealing that the change in the urinary microbiome might be associated with BCG treatment.

**Supplementary Information:**

The online version contains supplementary material available at 10.1007/s11255-025-04607-x.

## Introduction

Urinary bladder cancer is the ninth most common cancer worldwide with approximately 550,000 new cases diagnosed and 220,000 deaths every year [1–3]. Around 75% of patients with bladder cancer are non-muscle-invasive bladder cancer (NMIBC). Patients with high-risk NMIBC have a 60–70% chance of recurrence and 10–45% chance of progression to muscle-invasive or metastatic disease over 5 years. These non-muscle-invasive tumors can be classified as low grade or high grade and encompass multiple growth patterns, including papillary tumors and carcinoma in situ (CIS), a flattened layer of dysplastic cells that is presumed to represent a common precursor of muscle-invasive bladder cancer. In contrast, muscle-invasive bladder cancers have a relatively poor prognosis. NMIBC requires lifetime examination with regular cystoscopy and urinary cytology, making it one of the most expensive cancers to manage.

BCG is a species initiated after 230 passages of the pathogen *Mycobacterium bovis*. From the original pathogenic *M. bovis*, Albert Calmette and Camille Guérin successfully cultured a specific non-pathogenic bacillus that could protect against tuberculosis (TB). Not only for TB prevention but the potential of BCG was also investigated and applied in cancer treatment. In the 1970s, BCG was approved as an immunotherapeutic treatment for bladder cancer [4]. Until recently, current guidelines recommend the use of intravesical BCG in patients with intermediate- to high-risk NMIBC to reduce the risk of recurrence and progression, and it has been shown to be effective in reducing bladder cancer recurrence and disease progression [5]. Although BCG treatment works in the majority of cases, around 40% of bladder cancer patients do not respond well [6]. Understanding the impact of BCG on the urine microbiome may provide insight into BCG therapy in bladder cancer.

Traditionally, many would believe that urine is sterile; however, with the advancement of technology, the commensal bacteria profile in the urinary tract was identified in 2015 by Wolfe and Brubaker [7]. After that, the role of the urinary microbiome has been widely investigated. Recent reports described the urinary tract microbial communities could work in health maintenance but also in pathologies development after the alteration of bacterial composition [8]. Different urinary bacteria profiles could be associated with different diseases. Urinary tract infection (UTI) is one of the most common bacterial infections in humans. The majority of UTI is associated with Escherichia coli and some other gut-specific strains may also involve, such as Enterococcus and Staphylococcus [9]. The association between urinary microbiome and urothelial cancer has also been described recently. The result showed that specific micro-organisms, such as the genus *Pseudomonas, Anaerococcus, Schistosoma haematobium, or Fusobacterium* [10–12]*,* did over-colonize in bladder cancer patients’ urine. Moreover, the mechanism of BCG therapy on bladder cancer was also reported in some in vitro and in vivo studies. With the binding of fibronectin and α5β1 integrins, or the production of BCG-soluble factors such as Antigen85B, BCG induced cell cycle arrest and cell death [13–17]. However, the mechanism by which the bacteria in the BCG vaccine prevent cancer recurrence is incompletely elucidated.

Intravesical BCG is the most effective adjuvant treatment for intermediate- and high-grade non-muscle-invasive bladder cancer, and it has been proven to reduce bladder cancer recurrence and progression. Given the microbiological nature of BCG, its effect on urine microbiome alteration should also be investigated. In this cross-sectional study, urine of non-cancerous control, bladder cancer patients undergoing surveillance cystoscopy, and 1–2 year post-BCG group were examined. Modifying the urinary bacteria communities by BCG treatment may represent the BCG therapeutic response to bladder cancer. It may further improve our understanding of the mechanism of BCG as a bladder cancer therapy.

## Methods

### Study design

All patients were recruited from the Prince of Wales Hospital, Hong Kong SAR. Written informed consent was obtained before specimen collection (Supplementary). The study protocol was approved by the Joint Chinese University of Hong Kong-New Territories East Cluster Clinical Research Ethics Committee. The ethics approval reference number is CRE-2016.396.

A cross-sectional study of 9 non-bladder cancer patients (control), 13 recovered bladder cancer patients without BCG treatment (surveillance), and 12 1-2years post-BCG patients (post-BCG) were recruited. For all patients, a sterile container was provided for fresh urine collection. All urines were collected before flexible cystoscopy under sterile conditions. Once collected, urines were delivered to the laboratory within 1 h in the ice box and all urines were processed for bacteria isolation immediately.

### Urine bacteria DNA extraction

Urine samples were centrifuged at 8,000g for 10 min at 4C. The supernatant was discarded and pellets were resuspended in ice-cold PBS for washing. The second centrifugation was set at 10,000g for 10 min at 4C, with supernatant removal, and pellets were used for bacteria DNA extraction by QIAamp DNA Microbiome Kit (Qiagen, Hilden, USA). All extraction procedures followed the manufacturer's operating instructions and eluted DNA was quantified by NanoDrop™ 2000 Spectrophotometers (Thermo Fisher Scientific, Waltham, USA) and submitted for Next-Generation Sequencing.

### Library construction and 16S rRNA sequencing

The V4 regions of the 16S rRNA gene were amplified using specific primers 515F-806R as previously described [18]. All PCR reactions were performed with Phusion® High-Fidelity PCR Master Mix (New England Biolabs, Ipswich, USA) according to the manufacturer’s instructions. During the library preparation, combinatorial dual indexing was applied. The libraries were constructed using NEBNext® UltraTM DNA Library Prep Kit (Illumina, San Diego, USA), and then, paired-end sequencing was performed by Illumina NovaSeq 6000 Sequencing System (Illumina, San Diego, USA).

### Data processing using QIIME2

The raw reads were utilized to obtain clean reads after demultiplexing based on unique barcodes and removing primer sequences. Quality filtering on the clean reads was performed using the q2-dada2 plugin [19] implemented in QIIME2 (v2021.4) [20] as previously. The output file of dada2 was a feature table including all amplicon sequence variants (i.e., ASVs table).

For alpha-diversity and beta-diversity analyses, the sampling depth of 23,000 was used based on the dada2 feature table summary to retain more sequences per sample while excluding as few samples as possible. All alpha-diversity and beta-diversity indexes were computed using q2-diversity and plotted by the “microeco” R package (v0.5.2). The principal coordinate analysis (PCoA) plots based on Jaccard and unweighted UniFrac distances were displayed using the “microeco” R package. For taxonomic annotation, feature sequences were assigned to pre-trained naïve Bayes classifier trained on the SILVA 138 99% OTUs from the 515F/806R region of sequences [21]. And the taxonomic composition bar plot of samples among groups at the phyla level was generated using the “microeco” R package.

### Linear discriminant analysis effect Size (LEfSe) analysis and functional pathway predictions

Linear discriminant analysis (LDA) effect size (LEfSe) analysis [22] was used to identify significant taxonomic biomarkers between groups. The LEfSe analysis was calculated on the Galaxy instance of the Hutlab (https://huttenhower.sph.harvard.edu/galaxy) with default parameters. Taxonomic biomarkers with an LDA score threshold of > 2.0, *p* < 0.05 were considered as significant. Predictive metabolic function analysis of groups of bacteria was analyzed with Phylogenetic Investigation of Communities by Reconstruction of Unobserved States 2 (PICRUSt2, QIIME2-v2019.7 plugin) [23]. Differential metabolic pathways among groups were detected using LEfSe with an LDA score threshold of > 2.0, *p* < 0.05.

### Statistical analysis

The differences in clinical parameters among the three groups were computed with the Kruskal–Wallis test and Chi-square test for continuous and categorical data, respectively, by R (version 4.3.0). For alpha-diversity, Wilcoxon rank-sum test and multiple linear regression were adopted to calculate the statistical significance of differences among the groups. To compare microbial compositions between groups, beta-diversity was assessed based on Jaccard and unweighted UniFrac distance matrices. PERMANOVA and sex-stratified analysis were performed to calculate the significance of the differences in microbial compositions among groups and gender.

**Results**

### Demographic data

We divided the 34 patients into 3 groups: 12 patients who completed 12-month course of BCG instillation and at least 12 months from the 1st BCG instillation (**post-BCG**); 13 patients without BCG instillation (**surveillance**); and 9 patients who confirmed negative for bladder cancer on cystoscopy (**control**). Table 1 summarizes the details of patient characteristics. The groups were similar in baseline characteristics. Table S1-2 provide the detail information of the BCG treatment and cystoscopy timepoint, as well as the date of urine sample collection.

**Table 1:** Clinical characteristics of patients in control, surveillance, and post-BCG groups

	Control	Surveillance	Post-BCG	*p* value
	*n* = 9	*n* = 13	*n* = 12	
Age mean (median)	64(66)	70(73)	71(70)	0.11
BMI mean (median)	NA	36(37)	33(33)	0.25
Gender F	4	3	2	0.33
M	5	10	10	
Smoking Y	1	7	5	0.21
N	3	3	3	
NA	5	3	4	
DM Y	0	5	2	0.08
N	9	8	10	

# *DM*, diabetes mellitus, *F* female, *M* male, *NA* means missing data

### Taxonomic analysis of urinary microbiome

**Fig. 1:**
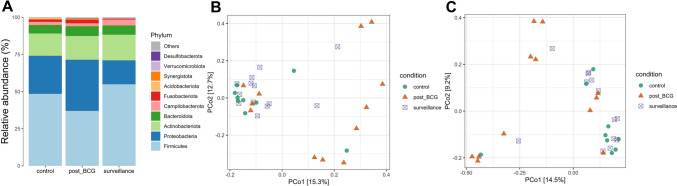
Taxonomic compositions of control, surveillance, and post-BCG groups. **A** The stacked bar plot showed the relative abundance of the top 10 microbial communities at the phyla level among the three groups. PCoA plot revealed the microbial differences among the three groups based on Jaccard (**B**) and unweighted UniFrac distances (**C**).

The taxonomical composition showed the major phyla across the groups were *Firmicutes, Proteobacteria, Actinobacteriota, and Bacteroidota* (Fig. 1A). Multiple linear regression and sex-stratified analyses demonstrated that there were no significant differences in alpha-diversity (such as Shannon index and InvSimpson) among control, surveillance, and post-BCG groups (all *p* > 0.05, Table S3-4, Figure S1). In contrast, multivariate PERMANOVA revealed that the effects of group on beta-diversity based on Jaccard and unweighted UniFrac distances were statistically significant (*p* value = 0.006/0.007, Table 2, 3), indicating that groups were the main driving factor for differences in community structure. Pairwise comparisons identified significant differences between control vs post-BCG (*p*-adjust = 0.029/0.017, Table 4, 5) and surveillance vs post-BCG (*p*-adjust = 0.006/0.017, Table 4, 5), indicating that the microbial composition of the post-BCG group was significantly different from that of the other two groups (Fig. 1B, C). There was no significant difference between control vs surveillance, suggesting that the community structures of these two groups were similar. Multivariate PERMANOVA suggested no main effect of gender on beta-diversity overall (*p* > 0.05, Table 2, 3), but sex-stratified analysis revealed a significant difference between the surveillance and post-BCG groups in men (p-adjust = 0.015/0.027, Table 6). This result may be related to the limited sample size in the female group, which led to insufficient statistical power to detect potential biological differences.

**Table 2:** Multivariate effects of group and gender on beta-diversity based on Jaccard distance assessed by PERMANOVA

Jaccard	Df	SumOfSqs	R2	F	Pr(> F)	Significance		
Condition	2	1.027	0.092	1.567	0.006	**		
Gender	1	0.320	0.029	0.975	0.492			
Residual	30	9.832	0.880	NA	NA			
Total	33	11.179	1	NA	NA			

**p < 0.01

**Table 3:** Multivariate effects of group and gender on beta-diversity based on unweighted UniFrac distance by PERMANOVA

Unweighted	Df	SumOfSqs	R2	F	Pr(> F)	Significance	
Condition	2	0.783	0.101	1.746	0.007	**	
Gender	1	0.217	0.028	0.968	0.471		
Residual	30	6.730	0.871	NA	NA		
Total	33	7.730	1	NA	NA		

**p < 0.01

**Table 4:** The pairwise PERMANOVA statistical results of beta-diversity based on Jaccard distance

Pairwise groups	measure	F	R2	p value	*p* adjust	Significance
control vs surveillance	Jaccard	0.990	0.047	0.451	0.451	
control vs post-BCG	Jaccard	1.724	0.083	0.019	0.029	*
surveillance vs post-BCG	Jaccard	1.865	0.075	0.002	0.006	**

*p adjust < 0.05; **p adjust < 0.01

**Table 5:** The pairwise PERMANOVA statistical results of beta-diversity based on unweighted UniFrac distance

Pairwise groups	Measure	F	R2	*p* value	p adjust	Significance
Control vs surveillance	Unweighted	1.060	0.050	0.334	0.334	
Control vs post-BCG	Unweighted	1.969	0.094	0.011	0.017	*
Surveillance vs post-BCG	Unweighted	2.015	0.081	0.006	0.017	*

*p adjust < 0.05

**Table 6:** Pairwise PERMANOVA of beta diversity index between groups stratified by gender. (*: *p* adjust < 0.05).

Gender	Groups	Measure	F	R2	*p*-value	*p*-adjust	Significance
Female	control vs surveillance	Jaccard	1.074	0.177	0.391	0.587	
Female	control vs post BCG	Jaccard	1.525	0.276	0.133	0.400	
Female	surveillance vs post BCG	Jaccard	0.966	0.244	0.600	0.600	
Male	control vs surveillance	Jaccard	1.100	0.078	0.304	0.304	
Male	control vs post BCG	Jaccard	1.150	0.081	0.227	0.304	
Male	surveillance vs post BCG	Jaccard	1.883	0.095	0.005	0.015	*
Female	control vs surveillance	unwei_unifrac	1.237	0.198	0.183	0.275	
Female	control vs post BCG	unwei_unifrac	1.847	0.316	0.067	0.200	
Female	surveillance vs post BCG	unwei_unifrac	0.920	0.235	0.600	0.600	
Male	control vs surveillance	unwei_unifrac	0.928	0.067	0.557	0.557	
Male	control vs post BCG	unwei_unifrac	1.171	0.083	0.250	0.375	
Male	surveillance vs post BCG	unwei_unifrac	1.873	0.094	0.009	0.027	*

### Taxonomic differences between groups

**Fig. 2:**
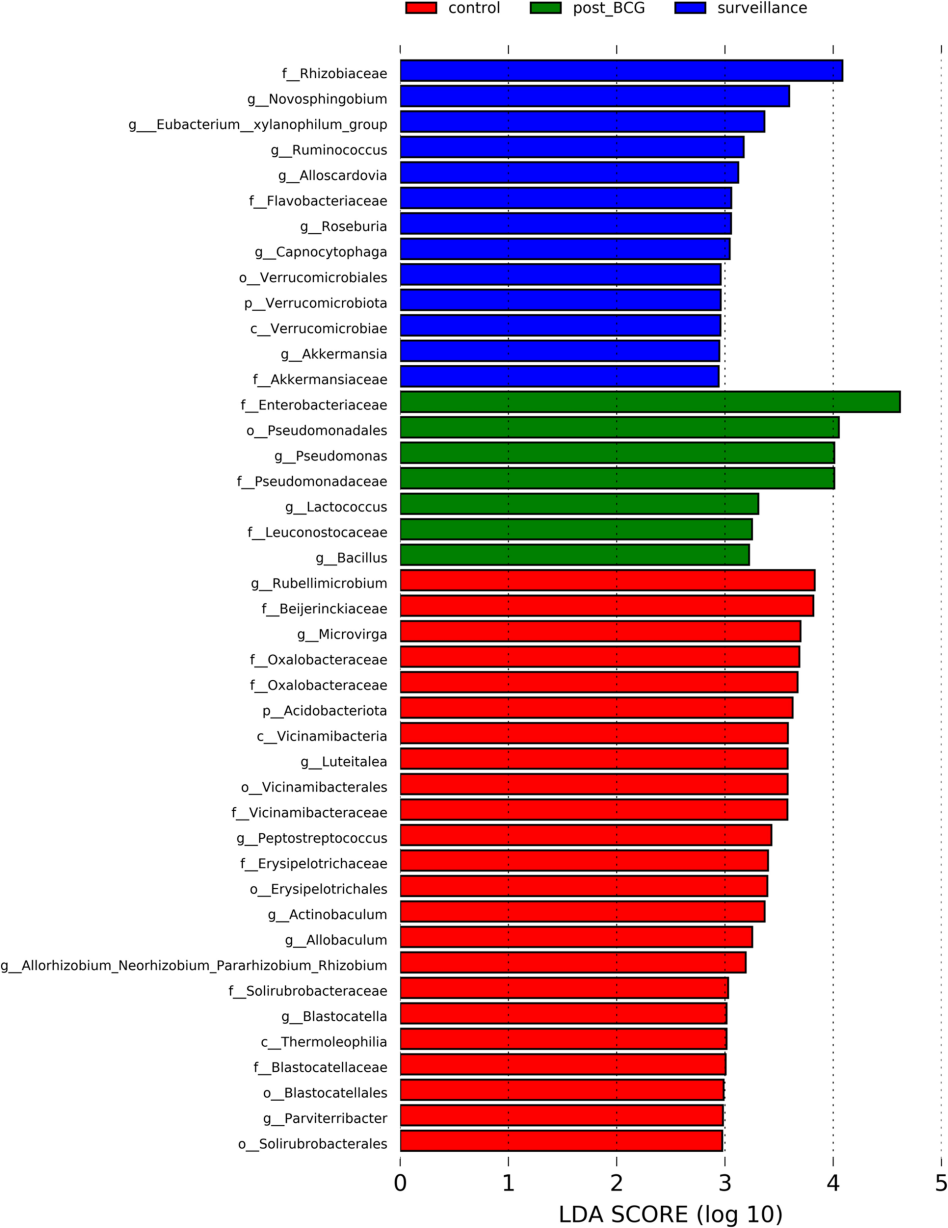
Differentially enriched microbiota. A histogram of LDA scores is shown as the result of LEfSe analysis for evaluating biomarkers with the statistical difference among groups. Differentially enriched microbiota was considered as biomarkers with an LDA score > 2 & *p* < 0.05

We further analyzed the taxonomic differences between groups with LEfSe. Surveillance group was enriched with genera *Novosphinogobium, Eubacterium xylanophilum, Ruminococcus, Alloscardovia, Roseburia, Capnocytophaga,* and *Akkermansia*. Whereas post-BCG group had higher abundance of genera *Pseudomonas, Lactococcus,* and *Bacillus* (Fig. 2).

**Fig. 3:**
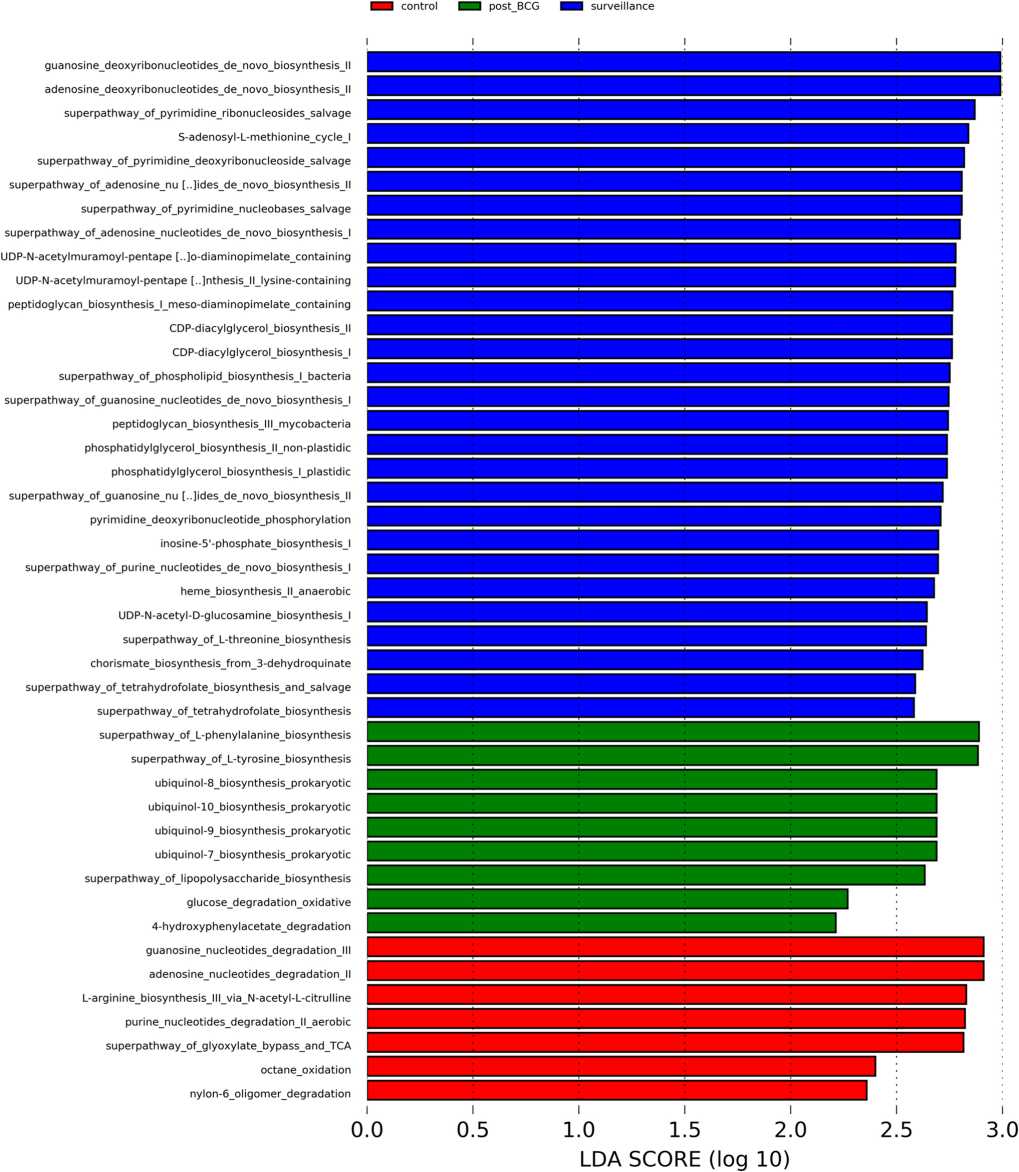
Differentially enriched pathways based on PICRUSt2. Differential functional pathways were assessed by LEfSe with an LDA score > 2 & *p* < 0.05

We predicted the functional pathways of urinary bacteria with PICRUSt2 and evaluated the result by LEfSe. The results showed that de novo biosynthesis pathways of different nucleotides (guanosine, adenosine, pyrimidine, and purine), bacteria cell wall biosynthesis (peptidoglycan), and amino acid biosynthesis (threonine) were enriched in the surveillance group. On the other hand, the superpathway of L-phenylalanine biosynthesis, superpathway of L-tyrosine biosynthesis, ubiquinol-7, 8, 9, 10 biosynthesis, superpathway of lipopolysaccharide biosynthesis, glucose degradation oxidative, and 4-hydroxyphenylacetate degradation were significantly enhanced in post-BCG group (Fig. 3).

**Discussion**

It was once believed that human urine and bladder are sterile. However, with the development of technology, the urinary microbiome evolved to become a potentially important factor influencing bladder cancer development and therapeutic responsiveness.

### Regarding surveillance group vs control group

Several studies have investigated the variety of bacteria in urine and demonstrated how microbial populations could influence the urological disease condition [24–26]. In our study, we showed that genus-level *Novosphingobium*, *Eubacterium xylanophilum, Ruminococcus, Alloscardovia, Roseburia, Capnocytophaga,* and *Akkermansia* were dominant in the bladder cancer surveillance group. Among all the above, only *Ruminococcus, Alloscardovia, and Akkermansia* have been reported in the urine microbiome.

Research have shown *Ruminococcus* in urine only in the context of chronic prostatitis/chronic pelvic pain syndrome and type 2 diabetic patients [27, 28]. While urinary *Ruminococcus* colonization was positively associated with urinary IL-8 (a pro-inflammatory cytokine) [27], its role in bladder cancer development is uncertain. On the other hand, *Alloscardovia omnicolens* correlated with urinary tract symptoms [29]. Urine microbial metagenomic study has also demonstrated enrichment of urinary *Alloscardovia* in patients with UTI. *Akkermansia* demonstrated its over-presentation in bladder cancer tissues and urine, it is also strongly associated with increased tumor burden [25]. Our findings also showed higher abundance of *Akkermansia* in bladder cancer.

The PICRUSt2 analysis demonstrated that the majority of the enriched pathways in the surveillance group were nucleotides biosynthesis and salvage pathways, and bacteria cell wall biosynthesis pathways. This implies that the bacteria in urine of these patients are highly proliferating. While the patients in surveillance group had no recurrence and their urine microbiome profile lacks commonly reported *Streptococcus* or *Fusobacterium*, this may reflect the stable disease condition of this cohort [12].

### Regarding post-BCG group vs control group

Intravesical BCG represents the standard-of-care for intermediate-risk and high-risk NMIBC. While BCG treatment could modulate the urine microbiome, it remains unsure what it brings to the final taxonomy and pathway mechanisms that helps with preventing recurrence or progression.

Our result demonstrated that genus *Pseudomonas, Lactococcus,* and *Bacillus* are dominant in urine of post-BCG group, while the previous reports suggested the anticancer potential of *Lactococcus* [30] and *Bacillus* [31]. Responders of intravesical BCG had relatively higher abundance of *Pseudomonas* in urine [32]. The potential therapeutic role of Pseudomonas may lie on its toxin. Oportuzumab monatox is a recombinant protein consisting of a pseudomonas toxin linked to a single-chain antibody that can bind EpCAM on the surface of bladder cancer cells. The whole payload is delivered specifically to cancer cells and induces cell death [33]. Therefore, enriched pseudomonas or factors secreted by pseudomonas are assumed to be toxic to bladder cancer cells.

Heidrich et al. used benign prostatic hyperplasia patients as the control group to investigate the impact of BCG therapy on the bladder microbiota. They demonstrated that BCG cannot persist in the bladder microbiota nor significantly alter its diversity and composition [34]. However, the control group was a different disease that might influence the microbiota itself. On the contrary, Boban et al. found that differences in microbiota composition before the start of BCG therapy between responders and non-responders to BCG therapy. Non-responders exhibited a 12 times higher abundance of genus *Aureispira* (*p* < 0.001), and, at the species level, a 27-fold lower abundance of *Negativicoccus succinivorans* (*p* < 0.001)[35]. While they focus on the prediction value of microbiota for BCG response, our study was focus on the impact of BCG on the microbiota changes in NMIBC patients.

Additionally, whole-metagenome shotgun sequencing (WMS) is considered superior to 16S rRNA sequencing for microbiota analysis [36], and we believe that technological advancements can enable more accurate detection of microbial flora in urine; however, the accessibility and cost of such technologies remain significant challenges.

Regarding the predicted functional pathways, L-phenylalanine biosynthesis superpathway, L-tyrosine biosynthesis superpathway, ubiquinols biosynthesis pathways, and superpathway of lipopolysaccharide (LPS) biosynthesis are significantly enhanced in our post-BCG group.

Phenylalanine and tyrosine serve as markers of immune activation, and their modulation by BCG immunotherapy on bladder cancer is further supported by our results [37]. Ubiquinol 7 demonstrated its antioxidant activity in egg yolk model [38]. For the superpathway of LPS biosynthesis, Han and colleagues reported that BCG lipopolysaccharide relates to 4 pathways and 10 gene ontology terms that intravesical BCG instillation can affect [39]. Being an agonist to Toll-like receptors (TLR), BCG can stimulate TLR2 and TLR4 through several bacterial cell wall components such as LPS. TLR agonists are promising immunostimulants and have a vital role in immune response induction, with significant immunotherapeutic ability against bladder cancer [40]. From our results, we speculate that BCG potentially modulates the urine microbiome profile and thus increases LPS biosynthesis and therefore may kill bladder cancer cells through the TLR-mediated immune responses.

### Limitations and conclusions

Our study is not without limitations. First of all, the sample size is small in our current analysis. Second, this is a cross-sectional study and we cannot make a cause–consequence relationship between treatment with BCG and the changes in the urine microbiome. We are planning for a prospective design by including a pre-BCG urine sample. Third, the “healthy” controls may have other conditions that influences urinary microbiome. Finally, we observed that not all urines from patients given BCG had a detectable bacteria profile, and therefore, the urinary bacteria DNA extraction method required optimization before proceeding to validations from a larger cohort.

To summarize our findings, BCG instillation favors the presentation of bacteria, such as *Pseudomonas, Lactococcus,* and *Bacillus*, and enriches potential antioxidant and anticancer pathways that enhance BCG therapy against bladder cancer. Although there are few reports describing the impact of BCG on the urinary microbiome, we are the first report to describe the enriched LPS biosynthesis pathway playing a role in BCG treatment in bladder cancer.

We believe that different bacteria are connected through physical and metabolic associations that present a balanced advantage to the entire urinary microbial community. Dissecting the urinary microbiome and its association with bladder microenvironments can assist to resolve this puzzle and open an opportunity for a new therapy, such as bacteriotherapy, and preventing severe adverse reactions by BCG treatment.

## Supplementary Information

Below is the link to the electronic supplementary material.

Supplementary file1 (DOCX 204 KB)

Supplementary file2 (DOCX 25 KB)

## Data Availability

The data that support the findings of this study are not openly available. The data can be made available from the corresponding author upon reasonable request strictly for research purpose.
