# Supplementary material for: A cross-sectional study on the effects of intravesical BCG on urinary microbiota in bladder cancer patients
Source: Int Urol Nephrol. 2025 Jun 30;58(1):67–76. doi: 10.1007/s11255-025-04607-x (PMC12783175; doi:10.1007/s11255-025-04607-x)
Supplement: Supplementary file 1 — Supplementary file1 (DOCX 204 KB) [file 11255_2025_4607_MOESM1_ESM.docx]

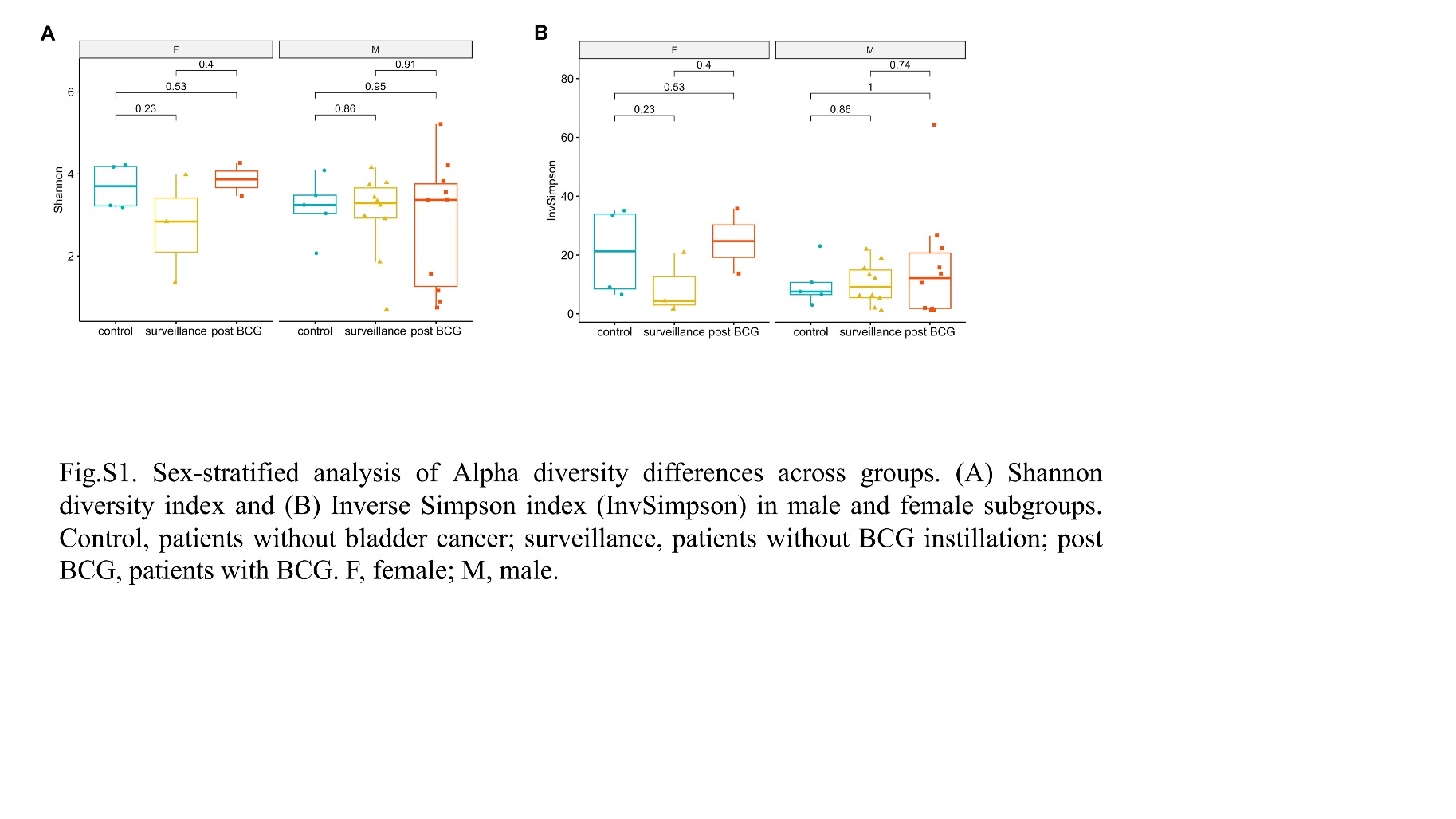


Fig.S1 Sex-stratified analysis of Alpha diversity differences across groups. (A) Shannon diversity index and (B) Inverse Simpson index (InvSimpson) in male and female subgroups. Control, patients without bladder cancer; surveillance, patients without BCG instillation; post BCG, patients with BCG. F, female; M, male.
