# Supplementary material for: A cross-sectional study on the effects of intravesical BCG on urinary microbiota in bladder cancer patients
Source: Int Urol Nephrol. 2025 Jun 30;58(1):67–76. doi: 10.1007/s11255-025-04607-x (PMC12783175; doi:10.1007/s11255-025-04607-x)
Supplement: Supplementary file 2 — Supplementary file2 (DOCX 25 KB) [file 11255_2025_4607_MOESM2_ESM.docx]

**Table S1. BCG treatment time of the post-BCG group.**

| Case# | Date of Urine Sample Collection | Date of First BCG treatment | Date of Last BCG treatment |
| --- | --- | --- | --- |
| BCG097 | 2020/12/11 | 2018/9/21 | 2019/8/30 |
| BCG116 | 2021/2/19 | 2019/10/8 | 2020/9/11 |
| BCG122 | 2021/3/26 | 2017/11/21 | 2018/11/6 |
| BCG038 | 2019/8/16 | 2018/9/21 | 2019/8/30 |
| BCG062 | 2019/12/27 | 2018/11/9 | 2020/1/16 |
| BCG058 | 2019/12/3 | 2018/12/6 | 2019/6/14 |
| BCG080 | 2020/6/23 | 2019/2/19 | 2020/4/2 |
| BCG031 | 2019/6/4 | 2019/4/4 | 2019/9/10 |
| BCG059 | 2019/12/3 | 2019/4/4 | 2019/9/10 |
| BCG004 | 2018/11/16 | 2018/9/21 | 2019/8/30 |
| BCG014 | 2019/2/22 | 2018/9/21 | 2019/8/30 |
| BCG023 | 2019/5/24 | 2018/9/21 | 2019/8/30 |

**Table S2. Cystoscopy treatment time.**

| Case# | Date of Urine Sample Collection | Group | Date of First cystoscopy | Date of Last cystoscopy |
| --- | --- | --- | --- | --- |
| BCG091 | 2020/11/27 | Control | 2020/11/27 |  |
| BCG099 | 2020/12/18 | Control | 2020/12/18 |  |
| BCG102 | 2020/12/18 | Control | 2020/12/18 |  |
| BCG105 | 2021/1/8 | Control | 2021/1/8 |  |
| BCG107 | 2021/1/8 | Control | 2021/1/8 |  |
| BCG110 | 2021/1/15 | Control | 2021/1/15 |  |
| BCG111 | 2021/1/15 | Control | 2021/1/15 |  |
| BCG114 | 2021/1/15 | Control | 2021/1/15 |  |
| BCG117 | 2021/3/12 | Control | 2021/3/12 |  |
| BCG089 | 2020/11/13 | surveillance | 2017/10/17 | 2021/3/12 |
| BCG090 | 2020/11/27 | surveillance | 2018/3/27 | 2020/11/27 |
| BCG093 | 2020/12/11 | surveillance | 2017/6/9 | 2020/12/11 |
| BCG095 | 2020/12/11 | surveillance | 2019/6/11 | 2020/12/11 |
| BCG096 | 2020/12/11 | surveillance | 2019/4/26 | 2021/6/11 |
| BCG098 | 2020/12/18 | surveillance | 2019/3/8 | 2020/12/18 |
| BCG100 | 2020/12/18 | surveillance | 2019/3/6 | 2020/12/18 |
| BCG101 | 2020/12/18 | surveillance | 2019/4/9 | 2021/6/18 |
| BCG103 | 2020/12/18 | surveillance | 2016/9/23 | 2021/6/18 |
| BCG109 | 2021/1/8 | surveillance | 2018/12/28 | 2021/7/9 |
| BCG113 | 2021/1/15 | surveillance | 2018/9/14 | 2021/7/16 |
| BCG115 | 2021/1/15 | surveillance | 2019/6/21 | 2021/7/30 |
| BCG118 | 2021/3/12 | surveillance | 2020/5/19 | 2021/6/11 |
| BCG097 | 2020/12/11 | post BCG | 2018/7/6 | 2021/6/11 |
| BCG116 | 2021/2/19 | post BCG | 2019/3/22 | 2021/8/17 |
| BCG122 | 2021/3/26 | post BCG | 2017/6/9 | 2021/3/26 |
| BCG038 | 2019/8/16 | post BCG | 2018/7/6 | 2021/6/11 |
| BCG062 | 2019/12/27 | post BCG | 2018/1/29 | 2021/4/9 |
| BCG058 | 2019/12/3 | post BCG | 2018/6/22 | 2021/8/3 |
| BCG080 | 2020/6/23 | post BCG | 2016/11/1 | 2021/6/15 |
| BCG031 | 2019/6/4 | post BCG | 2019/1/14 | 2021/8/13 |
| BCG059 | 2019/12/3 | post BCG | 2019/1/14 | 2021/8/13 |
| BCG004 | 2018/11/16 | post BCG | 2018/7/6 | 2021/6/11 |
| BCG014 | 2019/2/22 | post BCG | 2018/7/6 | 2021/6/11 |
| BCG023 | 2019/5/24 | post BCG | 2018/7/6 | 2021/6/11 |

| **Table S3. Multiple linear regression analysis of group and gender on Shannon index** | | | | |
| --- | --- | --- | --- | --- |
| Shannon index | Estimate | Std. Error | t value | Pr(>\|t\|) |
| (Intercept) | 6.240 | 1.717 | 3.635 | 0.002 |
| condition-post BCG | -0.462 | 0.602 | -0.767 | 0.454 |
| condition-surveillance | 0.368 | 0.617 | 0.597 | 0.559 |
| age | -0.041 | 0.028 | -1.460 | 0.164 |
| gender-M | 0.083 | 0.704 | 0.118 | 0.907 |
| smoking-Y | -0.642 | 0.504 | -1.274 | 0.221 |

| **Table S4. Multiple linear regression analysis of group and gender on Inverse Simpson index** | | | | |
| --- | --- | --- | --- | --- |
| InvSimpson index | Estimate | Std. Error | t value | Pr(>\|t\|) |
| (Intercept) | 38.054 | 16.990 | 2.240 | 0.040 |
| condition-post BCG | -2.698 | 5.953 | -0.453 | 0.657 |
| condition-surveillance | -1.954 | 6.104 | -0.320 | 0.753 |
| age | -0.236 | 0.275 | -0.858 | 0.403 |
| gender-M | -4.829 | 6.967 | -0.693 | 0.498 |
| smoking-Y | -5.320 | 4.991 | -1.066 | 0.302 |
